# Decreased expression of let-7c is associated with non-response of muscle-invasive bladder cancer patients to neoadjuvant chemotherapy

**DOI:** 10.18632/genesandcancer.103

**Published:** 2016-03

**Authors:** Ruth L. Vinall, Clifford G. Tepper, Alexandra A. Z. Ripoll, Regina F. Gandour-Edwards, Blythe P. Durbin-Johnson, Stanley A. Yap, Paramita M. Ghosh, Ralph W. deVere White

**Affiliations:** ^1^ Department of Urology, University of California, Davis, School of Medicine and Comprehensive Cancer Center, Sacramento, California, USA; ^2^ Department of Biochemistry and Molecular Medicine, University of California, Davis, School of Medicine and Comprehensive Cancer Center, Sacramento, California, USA; ^3^ Department of Pathology, University of California, Davis, School of Medicine and Comprehensive Cancer Center, Sacramento, California, USA; ^4^ Department of Public Health Sciences, University of California Davis, Davis, California, USA; ^5^ VA Northern California Health Care System, Mather, CA, USA; ^6^ California Northstate University College of Pharmacy, Elk Grove, CA, USA

**Keywords:** miRNA, neoadjuvant chemotherapy, muscle invasive bladder cancer, chemoresistance, let-7c

## Abstract

The identification and development of biomarkers which predict response of muscle invasive bladder cancer (MIBC) patients to neoadjuvant chemotherapy would likely increase usage of this treatment option and thereby improve patient survival rates. MiRNA array and qRT-PCR validation was used to identify miRNA which are associated with response to neoadjuvant chemotherapy. RNA was extracted from a total of 41 archival, fully annotated, MIBC patient diagnostic biopsies (20 chemo-responders and 21 non-responders (response is defined as > 5 year survival rate and being pT0 post-chemotherapy)). Microarray and qPCR identified let-7c as being differentially expressed in chemo-responder versus non-responder patients. Patients with higher let-7c expression levels had significantly higher odds of responding to chemotherapy (*p* = 0.023, OR 2.493, 95% CI 1.121, 5.546), and assessment of let-7c levels allowed for prediction of patient response (AUC 0.72, positive predictive value 59%). Decreased let-7c was associated with MIBC incidence (*p* < 0.001), and significantly correlated with other related miRNA including those that were not differentially expressed between responders and non-responders. The combined data indicate let-7c plays a role in mediating chemoresistance to neoadjuvant chemotherapy in MIBC patients, and is a modest, yet clinically meaningful, predictor of patient response.

## INTRODUCTION

There is an urgent need to improve survival rates for muscle-invasive bladder cancer (MIBC); the 5-year survival rate has changed very little over the last 20 years and is currently estimated to be between 30-50% [1–3]. Level 1 evidence supports the usage of platinum-based neoadjuvant chemotherapy for patients with MIBC; a randomized, double blind, multi-center clinical trial comparing neoadjuvant chemotherapy plus cystectomy to cystectomy alone reported a 77 month median survival and a 38% response rate for patients on neoadjuvant chemotherapy, *versus* a 46 month median survival and 15% response rate for cystectomy only (*p* < 0.001) [4]. While platinum-based neoadjuvant chemotherapy clearly improves patient outcome, response rates are still relatively low and this, combined with fear of treatment related toxicities, has resulted in limited usage of this treatment option. The ability to predict which patients will respond to platinum-based chemotherapy would likely increase usage and thereby improve MIBC survival rates. Other groups, including ours, have investigated whether *Tp53* status can predict patient response. Unfortunately, the data generated by these studies remain controversial due to methodological challenges associated with determining *Tp53* status, and *Tp53* status is not currently used to inform treatment decisions [5–9]. Plimack et al. recently reported defects in DNA repair genes is associated with response but this finding has not yet been prospectively validated [10]. To our knowledge, a validated biomarker does not exist to predict response of MIBC patients to neoadjuvant chemotherapy.

The goal of the current study was to determine whether alterations in miRNA expression levels are associated with response to platinum-based neoadjuvant chemotherapy, and whether miRNAs have the potential to be developed as predictive biomarkers. MiRNAs have several features that make them attractive biomarkers; their dysregulation has been shown to play a causative role in mediating carcinogenesis [11], they have unique expression profiles in different cancer types at different stages of disease [12], and they are relatively stable in both FFPE tissue and blood [13–15]. In addition, several recent studies have demonstrated that dysregulation of miRNA expression can promote chemoresistance in several cancers, including bladder cancer [16–18]. Tao et al., have demonstrated that inhibition of miR-21 expression sensitizes bladder cancer cell lines to doxorubicin treatment [17], and Kozinn et al., have correlated differences in miRNA expression levels with sensitivity of bladder cancer cell lines to gemcitabine [18]. We used miRNA array to identify the most differentially expressed miRNA in MIBC patients who responded *versus* did not respond to platinum-based chemotherapy (based on surgical resection post-chemotherapy and 5-year survival data; responders = patients with > 5 year survival rate, and who were pT0 post-chemotherapy), followed by quantitative real time PCR to validate differential expression of 9 of these miRNA in responders *versus* non-responders.

## RESULTS

### Patient characteristics

All patients had high grade cancers which were ≥ pT2 and all received neoadjuvant chemotherapy. There was no significant difference in median age between the responder (*n* = 21) and non-responder (*n* = 20) groups (p = 0.1187), and a similar number of males and females were represented in each group (Table 1). Balance of these patient characteristics between the groups is of note as both age and gender are prognostic risk factors for MIBC [19]. The following patient characteristics were also collected; carcinoma in situ (CIS) on TURBT, lymphovascular invasion on TURBT, hydronephrosis on CT, previous intravesical therapy (Table 1). All patient characteristics were balanced; there were no statistically significant differences between the groups.

**Table 1:** Patient characteristics

	(R) Responders	Median Age (range)	(NR) Non-responders	Median Age (Range)	*p*-value (R *vs* NR)
TOTAL	21	74 (54-82)	20	67 (49-81)	0.1187
Male (M)	18 (85.7%)	73 (54-82)	16 (80%)	67 (49-81)	0.2461
Female (F)	3 (14.29%)	77 (62.84)	4 (20%)	66.5 (59-71)	0.3189
Gemcitabine	16/17 (94.12%)		14/14 (100%)		0.399
Carboplatin/cisplatin	14/17 (82.35%)		14/14 (100%)		0.112
Taxol	10/17 (58.82%)		6/14 (42.86%)		0.396
BCG/MMC	4/21 (19.05%)		5/19 (26.32%)		0.6
CIS on TURBT	3/21 (14.29%)		3/19 (15.79%)		0.181
LVI on TURBT	4/21 (19.05%)		5/19 (26.32%)		0.218
Hydronephrosis on CT	6/20 (30%)		5/16 (31.25%)		0.255
Intravesical Therapy	4/20 (20%)		5/18 (27.78%)		0.229
p-value		0.61		0.8606	
(M vs F)					

Total participants = 41. All patients had high grade cancers which were ≥ pT2 and all were treated with a platinum-based neoadjuvant chemotherapy regimen. Comparator groups are non-responders to chemotherapy (*n* = 20) and responders (*n* = 21), (response is defined as > 5 year survival rate and being pT0 post-chemotherapy). There was no significant difference in patient characteristics between the two groups.

CIS = carcinoma in situ, TURBT = transurethral resection of bladder tumor, LVI = lymphovascular invasion, CT = computed tomography

### Microrray-based miRNA expression profiling of muscle invasive bladder cancers

**Figure 1:**
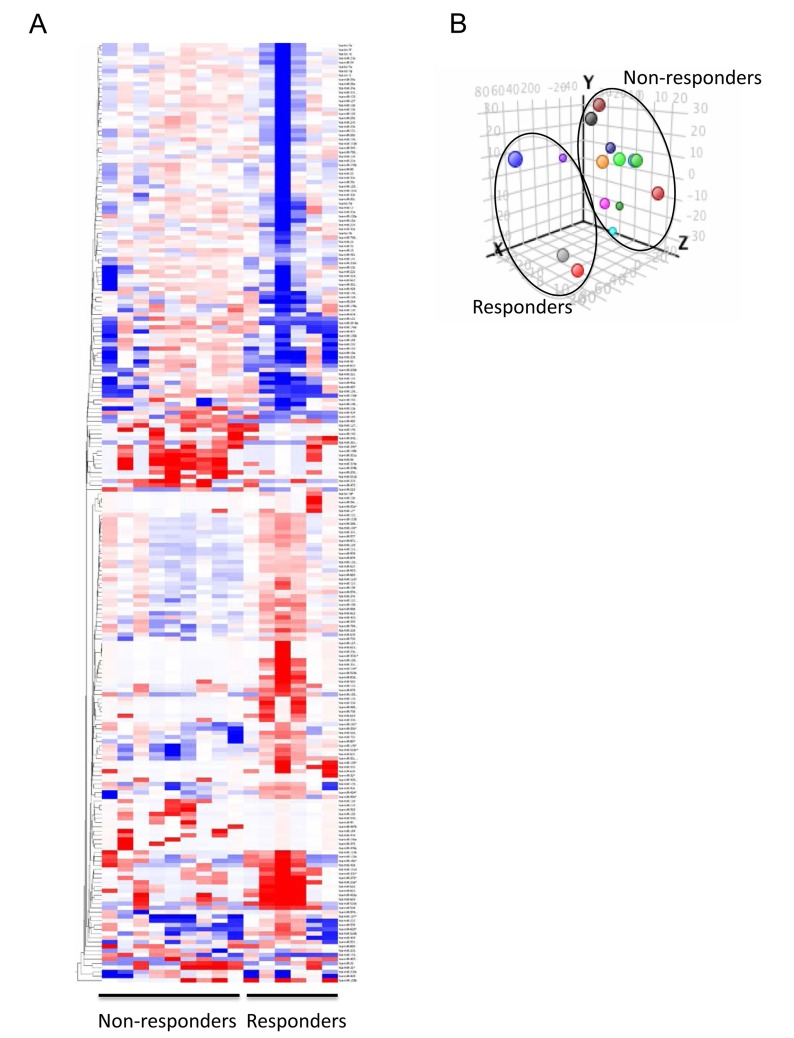
Comprehensive miRNA expression profiling of muscle invasive bladder cancer patients who responder *versus* did not respond to platinum-based chemotherapy **A.** Hierarchical clustering was performed on the data for 220 miRNAs that exhibited ≥1.5-fold change in expression in non-responsive tumors relative to the responsive group. The results are depicted as a heatmap with relative expression of each miRNA across the sample set ranging from −6.3 to +6.3 relative to the mean, red = high expression, blue = low expression. **B.** Principal components analysis (PCA) was performed on the array data and the results plotted based upon the identification of the three directions of maximal variation between the samples. The resulting graph depicts the overall similarities and differences between the miRNA populations from treated and untreated samples in an unbiased manner.

Fifteen of the 41 patients were randomly chosen for microarray-based miRNA expression profiling (6 chemotherapy responders, 9 non-responders). Comparison analysis of the array data was performed to filter for miRNAs having ≥1.5-fold change in expression between MIBC patients who responded (chemo-responder) *versus* those who did not (chemo-nonresponder) to neoadjuvant chemotherapy. This resulted in a set of 240 differentially-expressed miRNAs with 108 being elevated and 132 decreased in chemotherapy-responsive tumors relative to the non-responsive group (Supplementary Table 1). Hierarchical clustering was performed using the data for the 240 miRNAs and the results depicted as a heatmap (Figure 1A). The heatmap demonstrates that the two groups of samples have distinctive signatures, but at the same time reveals the existence of molecular heterogeneity in the samples. Principal components analysis (PCA) was performed on the array data and the results plotted based upon the identification of the three directions of maximal variation between the samples (Figure 1B). This analysis shows that differences in miRNA expression could be used to distinguish between responders and non-responders.

### Quantitative real time PCR analysis confirmed let-7c is significantly downregulated in chemo- non-responders compared to responders, and can predict patient response to neoadjuvant chemotherapy

**Figure 2:**
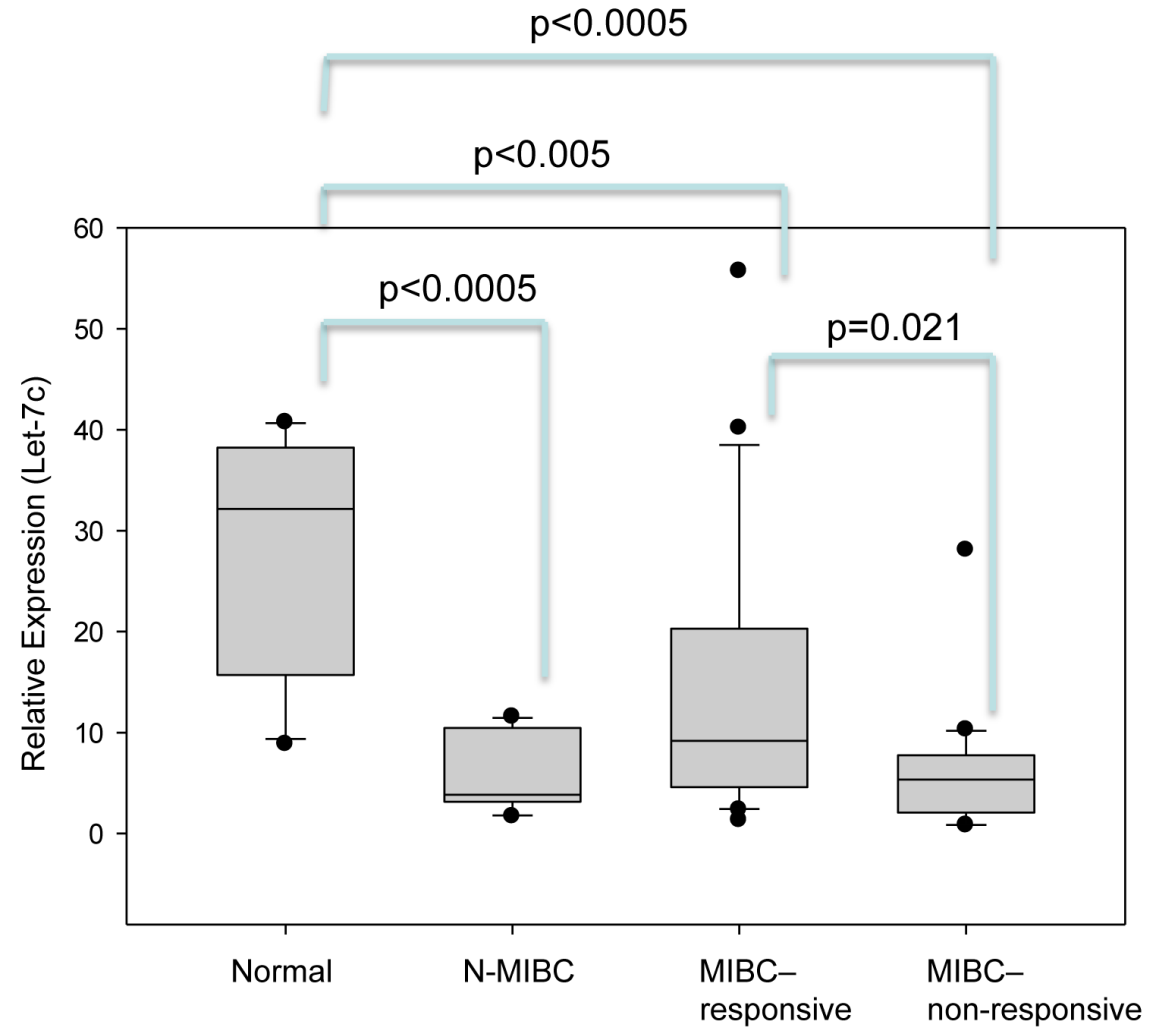
Decreased let-7c expression correlates with incidence and progression of muscle invasive bladder cancer, and with resistance to platinum-based chemotherapy Quantitative real time PCR determined that let-7c expression levels were significantly lower in patients with muscle invasive bladder cancer (MIBC) compared to patients without bladder cancer (normal). A further decrease in let-7c expression levels was observed between MIBC patients who did not respond to neoadjuvant chemotherapy (MIBC-non-responsive) but not in those who did respond (MIBC-responsive). Patients with non-invasive bladder cancer (N-MIBC) also expressed significantly lower levels of let-7c compared to patients without bladder cancer. There was no statistically significant difference in let-7c expression levels between MIBC and N-MIBC patients.

Ranking of miRNAs which were found to be differentially expressed by microarray analysis in decreasing order of fold change revealed that many of the most highly differentially expressed miRNAs have previously been linked to cancer progression and/or processes associated with cancer progression. Nine of these miRNA were chosen for validation by quantitative real-time PCR (qPCR); miR-34a, miR-15b, let-7c, let-7i, miR-10a, miR-16, miR-103, miR-106b, miR-200b. These miRNA were chosen not only based on their ranking in the miRNA array analysis but also by their linkage to putative and/or known targets which are associated with bladder cancer progression and/or chemoresistance (Table 2). The expression of these 9 miRNA were validated in tissues from all 41 patients described in Table 1. In the expanded set of 41 patients, out of the 9 miRNA selected for qPCR validation, only let-7c showed differential expression between patients who responded *versus* did not respond to neoadjuvant chemotherapy (Figure 2 and Table 3). A 42% decrease in let-7c was observed in non-responders vs responders (*p* = 0.021). Other miRNA that displayed large, but not significant, decreases include: miR-15b (41% decrease, *p* = 0.06), miR-200b (68% decrease, *p* = 0.062), miR-10a (51.5% decrease), miR-106b (30.7% decrease) (Table 3).

**Table 2:** Nine miRNA selected for validation by quantitative real time PCR

MiRNA Systematic Name	Array analysis, fold change (chemo-nonresponders versus responders)	MiRNA targets	Chromosomal band
**hsa-let-7c**	−6.491766	LIN28, Ras, HMGA2, c-myc, Bcl-xL	21q21.1
**hsa-miR-106b**	−5.045984	CCND1, E2F3, RBL1/2, WEE1	7q22.1
**hsa-miR-15b**	−5.901543	Cyclin E1, Bcl-2	3q25.33
**hsa-miR-103**	−4.588282	CCNE1, CDK2, CREB1	10q23.31
**hsa-let-7i**	−4.302098	E2F1, E2F3	12q14.1
**hsa-miR-16**	−2.735690	BCL2, MCL1, CCND1, WNT3A	13q14.2
**hsa-miR-34a**	−4.185326	Bcl-2, CCND1, CDK4/6, CREB, DLL1, E2F3, MET, c-MYC, SIRT-1, HMGA2, Notch1	1p36.22
**hsa-miR-10a**	−2.453648	NCOR2, SFRS1, Hoxd4	17q21.32
**hsa-miR-200b**	−1.835565	Ets-1, Suz12, cyclin D1, ZEB 1/2	1p36.33

Nine miRNA were chosen for validation by quantitative real-time PCR (qPCR) based on their ranking in the miRNA array analysis and their link to putative and/or known targets which are associated with bladder cancer progression and/or chemoresistance.

**Table 3:** Comparison of miRNA expression in patients with muscle invasive bladder cancer who responded *versus* did not respond to platinum-based chemotherapy

	RESPONDERS				NON-RESPONDERS				% change in median	*p*-value
miRNA	*n*	Median	Min	Max	*n*	Median	Min	Max		
miR-34a	21	7.832	2.294	52.902	20	7.651	1	25.924	−2.311	0.246
miR-15b	21	5.347	0.771	99.984	20	3.136	0.663	16.58	−41.350	0.06
Let-7c	21	9.171	1.293	55.683	20	5.338	0.773	28.073	−41.795	**0.021**
Let-7i	13	0.795	0.0403	6.813	13	0.694	0.0324	6.081	−12.704	0.758
miR-10a	13	4.483	0.918	22.105	12	2.171	0.561	14.041	−51.573	0.399
miR-16	13	8.727	1.37	58.062	13	8.756	0.659	19.736	0.331	0.837
miR-103	13	5.744	1.984	103	13	5.641	0.511	103	−1.793	0.372
miR-106b	13	18.055	3.523	257.248	13	26.059	1	100.931	30.715	0.837
miR-200b	21	42.405	3.365	442.268	20	13.629	1	87.283	−67.860	0.062

Out of the 9 miRNA selected for qPCR validation, only let-7c showed differential expression between patients who responded *versus* did not respond to platinum-based chemotherapy.

In univariate logistic regression analyses, patients with higher levels of let-7c had significantly higher odds of responding to neoadjuvant chemotherapy (*p* = 0.023, OR 2.493, 95% CI 1.121, 5.546, Table 4). The level of let-7c at which the predicted probability of response is 50% is 6.2. Thirteen out of 22 patients (59%) with let-7c levels above this cutoff responded to neoadjuvant chemotherapy, compared to 7 out of 18 (39%) patients with let-7c levels below this cutoff. For a cutoff of 6.2, the sensitivity of the classifier is 65% and the specificity is 55%, the positive predictive value is 0.59, and the negative predictive value is 0.61. The area under the curve (AUC) is 0.72, 95% CI (0.64, 0.79). These data indicate that let-7c is a fair predictor of response to chemotherapy. Higher odds of response were also observed with higher expression levels of miR-15b and miR-200b, but did not reach statistical significance (miR-15b; *p* = 0.058, OR 2.012, 95% CI 0.968, 4.179, miR-200b; *p* = 0.059, OR = 1.640, 95% CI = 0.976, 2.758).

**Table 4:** Univariate logistic regression analysis results

Marker	*n*	Odds Ratio	95% CI for Odds Ratio	*p*-value
miR-34a	41	1.803	(0.786, 4.137)	0.159
miR-15b	40	2.012	(0.968, 4.179)	0.058
Let-7c	40	2.493	(1.121, 5.546)	**0.023**
Let-7i	26	1.049	(0.688, 1.600)	0.822
miR-10a	25	1.546	(0.705, 3.390)	0.271
miR-16	26	1.352	(0.618, 2.955)	0.444
miR-103	23	1.795	(0.701, 4.596)	0.217
miR-106b	26	1.087	(0.597, 1.980)	0.782
miR-200b	36	1.640	(0.976, 2.758)	0.059

* Observed response rate is 100% or 0% in either marker+ or marker-subset, leading to an estimate of 0 or Inf for the odds ratio and a 95% confidence interval of (0, Inf).

In univariate logistic regression analyses, patients with higher levels of let-7c had significantly higher odds of responding to neoadjuvant chemotherapy (*p* = 0.023, OR 2.493, 95% CI 1.121, 5.546).

### Let-7c expression is higher in normal bladder compared to non-muscle invasive, and muscle-invasive bladder cancer

Since let-7c levels were higher in chemo-responsive bladder cancer compared to non-responsive patients, we investigated the levels of this miRNA in the normal bladder (normal, *n* = 10) as well as non-muscle invasive bladder cancer (N-MIBC, *n* = 10)) (Figure 2). Significantly, let-7c expression in non-tumor tissue was elevated 4.76-fold compared to all bladder cancer tissues (both MIBC and N-MIBC, *p* < 0.001). There was a significant difference in let-7c expression between normal bladder and N-MIBC (*p* < 0.0005, 95% CI 10.46, 34.13), and between normal and MIBC (MIBC responder; *p* < 0.005, 95% CI 4.279, 24.78, MIBC non-responder; *p* < 0.0005, 95% CI 12.04, 32.54), but no significant difference in let-7c expression between N-MIBC and MIBC (Figure 2). The loss of this miRNA in N-MIBC indicates reduction of let-7c expression may play an important role in driving BC initiation as well as mediating chemoresistance to platinum-based chemotherapy.

### Concomitant changes in miRNA expression in MIBC

**Figure 3:**
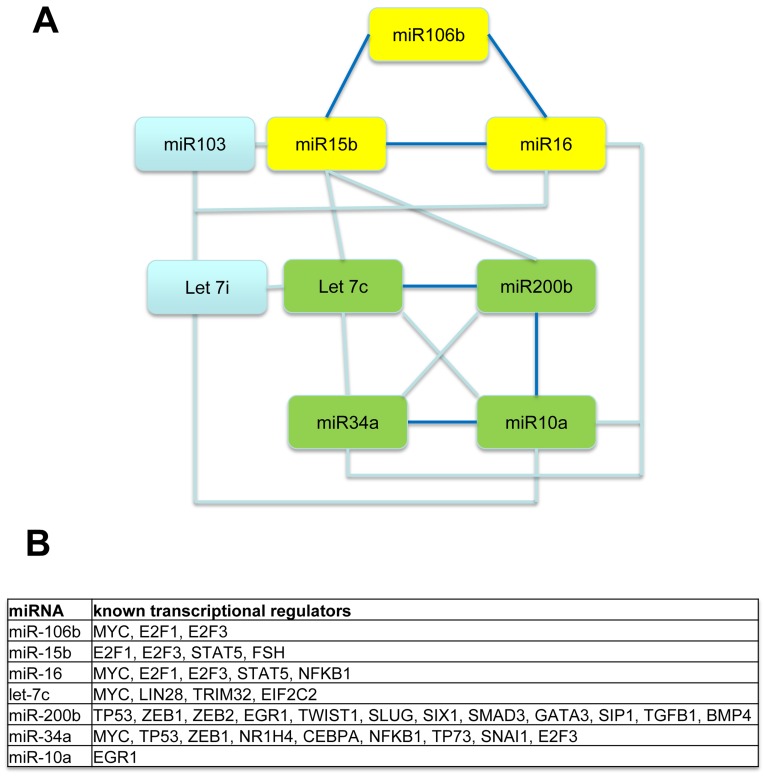
Concomitant changes in miRNA expression in individual patients The expression of several miRNAs trended together in terms of their relative expression levels **A**., and statistically significant correlations in expression were observed for miR-106b, miR-15b, and miR-16, and for let-7c, miR-200b, miR-34a, and miR-10a (please refer to Table 5 for correlation data). Use of the Transmir database revealed that expression of these grouped miRNA can be controlled by several of the same transcriptional regulators **B**., indicating this may be a mechanism of co-regulation.

As several of the miRNAs analyzed in this study target the same molecules and/or same signaling pathways (Table 2), we rationalized there may be coordinated control of their expression. Correlations in expression were observed for several of the miRNAs (Table 5). Figure 3A shows a graphic of these data indicating which miRNA trended most closely with each other; miR-106b, miR-15b, and miR-16 trended together, as did let-7c, miR-200b, miR-34a, and miR-10a. Utilization of the Transmir database revealed that the expression of these grouped miRNA can be controlled by several of the same transcription factors (Figure 3B). For example, E2F1 and E2F3 are transcription factors that can control expression of miR-106b, miR-15b, and miR-16. These data suggest that coordinated control of some miRNA expression may be important in determining patient response to chemotherapy.

**Table 5:** Concomitant changes in miRNA expression in individual patients

	Let-7c	Let-7i	miR-10a	miR-16	miR-103	miR-106b	miR-200b	
**miR-34a**	0.48		0.735	0.418			0.347	Coefficient
	0.00173		2.86E-05	0.0338			0.038	*P*-value
	40		25	26			36	count
**miR-15b**	0.376		0.493	0.952	0.5	0.8	0.534	Coefficient
	0.0169		0.0123	8.07E-14	0.0151	9.33E-07	0.000805	*P*-value
	40		25	26	23	26	36	count
								
**Let-7c**		0.437	0.442				0.79	Coefficient
		0.0257	0.0268				1.03E-08	*P*-value
		26	25				36	count
								
**Let-7i**					0.42			Coefficient
					0.0462			*P*-value
					23			count
								
**miR-10a**				0.467			0.709	Coefficient
				0.0185			0.000324	*P*-value
				25			21	count
**miR-16**								
					0.459	0.801		Coefficient
					0.0275	8.63E-07		*P*-value
					23	26		count

## DISCUSSION

Level 1 evidence clearly demonstrates neoadjuvant chemotherapy confers survival benefit to MIBC patients [4], however, it is underutilized [20]. The main reasons for this underutilization are the relatively low response rate of MIBC patients to neoadjuvant chemotherapy (currently ~50%) and the treatment regimen can cause significant toxicity. The latter is of particular importance for this patient population as many MIBC patients are older and have co-morbidities. The ability to predict patient response would likely increase usage of neoadjuvant chemotherapy and thereby improve MIBC survival rates. Our study identifies an association between let-7c expression and response of patients with MIBC to platinum-based neoadjuvant chemotherapy, and suggest let-7c may be able to predict patient response. Although let-7c has only a modest ability to predict response of MIBC patients to neoadjuvant chemotherapy (positive predictive value of 59%), we believe this may be sufficient to help guide treatment decisions and promote increased usage of neoadjuvant therapy. To our knowledge, this is the first patient study to identify let-7c as a potential predictive marker of response to neoadjuvant chemotherapy. A major benefit of assessing miRNA expression is that miRNA can be measured with great accuracy and reproducibility in patient specimens, including blood and urine. Multiple clinical studies have demonstrated that miRNA have the potential to be used as diagnostic, prognostic, and predictive biomarkers [21, 22].

*In vitro* studies in other cancer types have demonstrated an association between let-7c and chemoresistance [23–25]. For example, hepatoma cell lines that are resistant to chemotherapy express low levels of let-7c [26]. Forced expression of let-7c in these cells sensitizes them to sorafenib treatment through downregulation of Bcl-xL expression. Other validated targets of let-7c include LIN28, c-myc, H-Ras and HMGA2 [27–29]. All of these molecules can potentially play a role in mediating cancer initiation, progression and/or chemoresistance; Bcl-xL promotes cell survival, LIN28 regulates self-renewal of stem cells and is able to activate the PI3K-mTOR pathway, c-myc, H-Ras and HMGA2 all drive cell proliferation. *In vitro* studies are needed to determine whether let-7c can target these molecules in bladder cancer cells. H-Ras and HMGA2 are of particular interest because expression of these molecules is elevated in bladder cancer patients, and elevated H-Ras expression has been linked to worse patient outcome [30].

Several recent studies have established a link between let-7c expression and bladder cancer progression and/or survival, however, these studies were not specific to patients treated with neoadjuvant chemotherapy and some studies included N-MIBC patients [31–34]. For example, Itesako et al. reported let-7c expression is lower in bladder cancer patients (both MIBC and N-MIBC) compared to normal controls [32] (an association which we also observed). Xu et al. showed decreased let-7c expression is associated with MIBC patient survival, however, they excluded patients treated with neoadjuvant chemotherapy from their study [33]. Zhou et al. showed an association between let-7c expression and bladder cancer patient outcome, however, all MIBC cancer patients were included in their analysis, not only those treated with neoadjuvant chemotherapy [34]. The combined data underscore the importance of let-7c in bladder carcinogenesis and determining patient outcome as well as suggest let-7c may impact pathways which promote bladder cancer initiation and progression as well as response to chemotherapy. Again, *in vitro* analyses will be necessary to determine which molecules and pathways can be targeted by let-7c in bladder cancer cells.

It is noteworthy that while statistical significance was not achieved for the other 8 of the 9 miRNA assessed by qPCR, a median decrease of >40% in expression in miR-15b, miR-10a, and miR-200b was observed in non-responders compared to responders, and miR-106b showed a >40% increase. Such large differences in median expression indicate these miRNA may play a key role in response to chemotherapy in a subset of MIBC patients. It is also noteworthy that changes in expression levels of several miRNA trended together in individual patient samples, suggesting that control of their expression is coordinated. Coordinated control of miRNA expression is important in many biological systems to allow for the regulation of complex cellular processes [35], and can occur through genomic clustering, epigenetic regulation, or regulation by a shared transcription factor. Use of the Transmir database [36] confirmed that the latter is possible in our system. The miRNA whose expression trended together in our study share several of the same transcriptional regulators, for example, several can be regulated by E2F1 and E2F3. As both of these transcription factors act downstream of Rb, and Rb mutations are frequent in MIBC [37], it is possible that Rb mutation may be one way by which the dysregulation of expression of these miRNA occurs.

In summary, our combined data demonstrate that there is an association between let-7c and patient resistance to chemotherapy, and suggest let-7c may be a useful predictor of MIBC patient response to neoadjuvant chemotherapy. *In vitro* analysis will be needed to identify which molecules and pathways let-7c targets and the mechanisms by which decreased let-7c expression can mediate increased rates of MIBC patient survival. A major limitation of this study was sample size. Larger and prospective studies are needed to validate our findings.

## MATERIALS AND METHODS

### Ethics statement

Investigation has been conducted in accordance with the ethical standards and according to the Declaration of Helsinki and according to national and international guidelines and has been approved by the authors’ institutional review board.

The order of the various sections below describe the work flow of events during this analysis.

### Patient samples

A total of 41 formalin-fixed, paraffin-embedded (FFPE) bladder tumor samples were obtained with IRB consent from MIBC patients *prior* to treatment with platinum-based neoadjuvant chemotherapy. Twenty-one of these patients subsequently responded to treatment and twenty did not (based on surgical resection post-chemotherapy and 5-year survival data; responders = patients with > 5 year survival rate and who were pT0 post-chemotherapy). Ten samples from patients with non-muscle invasive bladder cancer (N-MIBC, median age 62.5) and ten ‘normal’ patient samples (median age 62) were also analyzed.

### RNA and DNA extraction

For isolation of RNA and DNA, 15-uM unstained sections were cut (5 sections for RNA isolation, 1 section for DNA isolation). The tumor area was outlined by a pathologist (R.G.E.) on an H&E stained section and matching areas on the unstained 15-uM sections removed using a scapel blade. RNA and DNA were isolated using Qiagen miRNeasy FFPE kits and QIAamp DNA FFPE tissue kits, respectively (Qiagen, Valencia, CA). DNA and RNA concentration and purity were assessed using a NanoDrop 2000 spectrophotometer.

### miRNA array analysis

Fifteen patient samples (6 responders, 9 non-responders) were used for miRNA array analyses. Comprehensive miRNA expression profiling was performed with Agilent Human miRNA Microarrays (Version 3) [38],[39] per manufacturer's protocol. Data analysis was performed with GeneSpring GX11 software (Agilent Technologies). Principal component analysis (PCA) was conducted as an unbiased method of exploratory analysis in order to visualize the overall differences and similarities between the miRNA populations derived from groups of tumors having different clinical parameters. Comparison analysis was then performed in order to identify the miRNAs that were *1)* differentially expressed (1.5-fold) in treatment-naïve bladder tumors that were subsequently classified as either chemotherapy-responsive or non-responsive and *2)* differentially-expressed between post-therapy recurrent and chemotherapy-naïve tumors. Hierarchical clustering of the data was utilized to identify patterns of miRNA expression and groups of co-expressed miRNAs.

### Quantitative real time PCR analysis

The expression of 9 miRNA (miR-34a, miR-15b, let-7c, let-7i, miR-10a, miR-16, miR-103, miR-106b, miR-200b) was analyzed in all 41 patient samples by quantitative real time RT-PCR (qPCR). MiRNA expression was assessed using pre-designed TaqMan primer/probes sets in combination with the TaqMan MicroRNA Reverse Transcription and Universal PCR Master Mix (no AmpErase UNG) kits as per manufacturer's protocol (Applied Biosystems, Foster City, CA) using the Applied Biosystems 7000HT PCR machine and associated software. Analysis of U6 expression was used to normalize miRNA expression between patient samples.

### Statistical analysis

Mann-Whitney unpaired analysis was used to assess differences in gender, and age between the responder and nonresponder groups. Wilcoxon signed rank analysis was used to assess differences in miRNA expression between the responder and non-responder groups. Mann-Whitney paired analysis was used to assess differences in miRNA expression in pre-*versus* post-chemotherapy specimens. The relationship between miRNA levels and response to chemotherapy was assessed by univariate logistic regression analyses. Data on miRNA levels were log transformed prior to logistic regression analyses in order to more closely satisfy model assumptions. ANOVA in combination with Tukey's multiple comparisons post-hoc analysis (GraphPad Prism) was used to compare relative let-7c expression in normal, non-muscle invasive and muscle invasive bladder cancer specimens.

## SUPPLEMENTARY FIGURE AND TABLE



## References

[1] Rene NJ, Cury FB, Souhami L (2009). Conservative treatment of invasive bladder cancer. Current oncology.

[2] Turkolmez K, Tokgoz H, Resorlu B, Kose K, Beduk Y (2007). Muscle-invasive bladder cancer: predictive factors and prognostic difference between primary and progressive tumors. Urology.

[3] Kluth LA, Black PC, Bochner BH, Catto J, Lerner SP, Stenzl A (2015). Prognostic and Prediction Tools in Bladder Cancer: A Comprehensive Review of the Literature. European urology.

[4] Grossman HB, Natale RB, Tangen CM, Speights VO, Vogelzang NJ, Trump DL (2003). Neoadjuvant chemotherapy plus cystectomy compared with cystectomy alone for locally advanced bladder cancer. N Engl J Med.

[5] Grossman HB, Cordon-Cardon S CR, Waldman FM, Tangen C, Crawford ED (2002). SWOG 9458: Evaluation of Ki-67, p53 and angiogenesis in patients registered to SWOG 9710. International Journal of Cancer.

[6] Grossman HB, Tangen CM, Cordon-Cardo C, Cote R, Waldman FM, De Vere White RW (2006). Evaluation of Ki67, p53 and angiogenesis in patients enrolled in a randomized study of neoadjuvant chemotherapy with or without cystectomy: a Southwest Oncology Group Study. Oncol Rep.

[7] Qureshi KN, Griffiths TR, Robinson MC, Marsh C, Roberts JT, Hall RR (1999). TP53 accumulation predicts improved survival in patients resistant to systemic cisplatin-based chemotherapy for muscle-invasive bladder cancer. Clin Cancer Res.

[8] Sarkis AS, Bajorin DF, Reuter VE, Herr HW, Netto G, Zhang ZF (1995). Prognostic value of p53 nuclear overexpression in patients with invasive bladder cancer treated with neoadjuvant MVAC. J Clin Oncol.

[9] Williams SG, Gandour-Edwards R, Deitch AD, Toscano S, Fan JJ, Sternberg CN (2001). Differences in gene expression in muscle-invasive bladder cancer: a comparison of Italian and American patients. Eur Urol.

[10] Plimack ER, Dunbrack RL, Brennan TA, Andrake MD, Zhou Y, Serebriiskii IG (2015). Defects in DNA Repair Genes Predict Response to Neoadjuvant Cisplatin-based Chemotherapy in Muscle-invasive Bladder Cancer. Eur Urol.

[11] Seven M, Karatas OF, Duz MB, Ozen M (2014). The role of miRNAs in cancer: from pathogenesis to therapeutic implications. Future oncology.

[12] Cortez MA, Ivan C, Zhou P, Wu X, Ivan M, Calin GA microRNAs in cancer: from bench to bedside. Adv Cancer Res.

[13] Mraz M, Malinova K, Mayer J, Pospisilova S (2009). MicroRNA isolation and stability in stored RNA samples. Biochem Biophys Res Commun.

[14] Kruhoffer M, Dyrskjot L, Voss T, Lindberg RL, Wyrich R, Thykjaer T (2007). Isolation of microarray-grade total RNA, microRNA, and DNA from a single PAXgene blood RNA tube. J Mol Diagn.

[15] Brase JC, Wuttig D, Kuner R, Sultmann H Serum microRNAs as non-invasive biomarkers for cancer. Mol Cancer.

[16] Allen KE, Weiss GJ Resistance may not be futile: microRNA biomarkers for chemoresistance and potential therapeutics. Mol Cancer Ther.

[17] Tao J, Lu Q, Wu D, Li P, Xu B, Qing W microRNA-21 modulates cell proliferation and sensitivity to doxorubicin in bladder cancer cells. Oncol Rep.

[18] Kozinn SI, Harty NJ, Delong JM, Deliyiannis C, Logvinenko T, Summerhayes IC MicroRNA Profile to Predict Gemcitabine Resistance in Bladder Carcinoma Cell Lines. Genes Cancer.

[19] Shariat SF, Zippe C, Ludecke G, Boman H, Sanchez-Carbayo M, Casella R (2005). Nomograms including nuclear matrix protein 22 for prediction of disease recurrence and progression in patients with Ta, T1 or CIS transitional cell carcinoma of the bladder. J Urol.

[20] David KA, Milowsky MI, Ritchey J, Carroll PR, Nanus DM (2007). Low incidence of perioperative chemotherapy for stage III bladder cancer 1998 to 2003: a report from the National Cancer Data Base. J Urol.

[21] Cheng G Circulating miRNAs: Roles in cancer diagnosis, prognosis and therapy. Adv Drug Deliv Rev.

[22] Jeffrey SS (2008). Cancer biomarker profiling with microRNAs. Nat Biotechnol.

[23] Sugimura K, Miyata H, Tanaka K, Hamano R, Takahashi T, Kurokawa Y Let-7 expression is a significant determinant of response to chemotherapy through the regulation of IL-6/STAT3 pathway in esophageal squamous cell carcinoma. Clin Cancer Res.

[24] Li Y, VandenBoom TG, Kong D, Wang Z, Ali S, Philip PA (2009). Up-regulation of miR-200 and let-7 by natural agents leads to the reversal of epithelial-to-mesenchymal transition in gemcitabine-resistant pancreatic cancer cells. Cancer Res.

[25] Cui SY, Huang JY, Chen YT, Song HZ, Feng B, Huang GC Let-7c Governs the Acquisition of Chemo- or Radioresistance and Epithelial-to-Mesenchymal Transition Phenotypes in Docetaxel-Resistant Lung Adenocarcinoma. Mol Cancer Res.

[26] Shimizu S, Takehara T, Hikita H, Kodama T, Miyagi T, Hosui A The let-7 family of microRNAs inhibits Bcl-xL expression and potentiates sorafenib-induced apoptosis in human hepatocellular carcinoma. J Hepatol.

[27] Johnson SM, Grosshans H, Shingara J, Byrom M, Jarvis R, Cheng A (2005). RAS is regulated by the let-7 microRNA family. Cell.

[28] Gomez CR, Kosari F, Munz JM, Schreiber CA, Knutson GJ, Ida CM Prognostic value of discs large homolog 7 transcript levels in prostate cancer. PLoS One.

[29] Mayr C, Hemann MT, Bartel DP (2007). Disrupting the pairing between let-7 and Hmga2 enhances oncogenic transformation. Science.

[30] Castillo-Martin M, Domingo-Domenech J, Karni-Schmidt O, Matos T, Cordon-Cardo C Molecular pathways of urothelial development and bladder tumorigenesis. Urol Oncol.

[31] Canturk KM, Ozdemir M, Can C, Oner S, Emre R, Aslan H (2014). Investigation of key miRNAs and target genes in bladder cancer using miRNA profiling and bioinformatic tools. Mol Biol Rep.

[32] Itesako T, Seki N, Yoshino H, Chiyomaru T, Yamasaki T, Hidaka H (2014). The microRNA expression signature of bladder cancer by deep sequencing: the functional significance of the miR-195/497 cluster. PLoS One.

[33] Xu Z, Yu YQ, Ge YZ, Zhu JG, Zhu M, Zhao YC (2015). MicroRNA expression profiles in muscle-invasive bladder cancer: identification of a four-microRNA signature associated with patient survival. Tumour Biol.

[34] Zhou H, Tang K, Xiao H, Zeng J, Guan W, Guo X (2015). A panel of eight-miRNA signature as a potential biomarker for predicting survival in bladder cancer. J Exp Clin Cancer Res.

[35] Shalgi R, Lieber D, Oren M, Pilpel Y (2007). Global and local architecture of the mammalian microRNA-transcription factor regulatory network. PLoS Comput Biol.

[36] Wang J, Lu M, Qiu C, Cui Q TransmiR: a transcription factor-microRNA regulation database. Nucleic Acids Res.

[37] Ziebold U, Lee EY, Bronson RT, Lees JA (2003). E2F3 loss has opposing effects on different pRB-deficient tumors, resulting in suppression of pituitary tumors but metastasis of medullary thyroid carcinomas. Mol Cell Biol.

[38] Hughes TR, Mao M, Jones AR, Burchard J, Marton MJ, Shannon KW (2001). Expression profiling using microarrays fabricated by an ink-jet oligonucleotide synthesizer. Nat Biotechnol.

[39] Wang H, Ach RA, Curry B (2007). Direct and sensitive miRNA profiling from low-input total RNA. RNA.

