# Supplementary material for: Decreased expression of let-7c is associated with non-response of muscle-invasive bladder cancer patients to neoadjuvant chemotherapy
Source: Genes Cancer. 2016 Mar;7(3-4):86–97. doi: 10.18632/genesandcancer.103 (PMC4918947; doi:10.18632/genesandcancer.103)
Supplement: Supplementary file 1 [file ganc-07-086-s001.pdf]

Decreased expression of let-7c is associated with non-response of muscle-invasive bladder cancer patients to neoadjuvant chemotherapy – Vinall et al

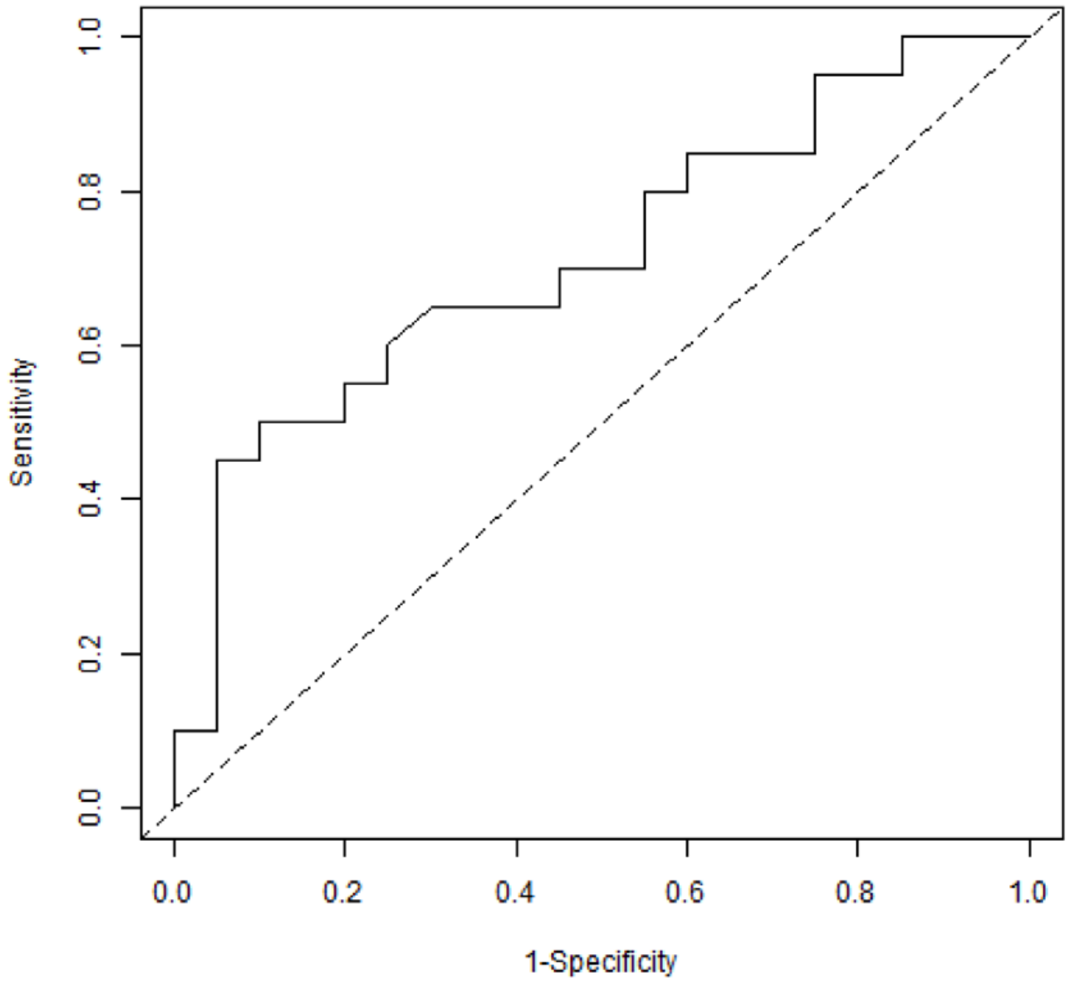

Supplementary Figure 1: Receiver operating curve (ROC) plot for classifier based on let-7c. The area under the ROC curve is 0.72. The level of let-7c at which the predicted probability of response is 50% is 6.2. Thirteen out of 22 patients (59%) with let-7c levels above this cutoff responded to neoadjuvant chemotherapy, compared to 7 out of 18 (39%) patients with let-7c levels below this cutoff. For a cutoff of 6.2, the sensitivity of the classifier is 65% and the specificity is 55%.

Supplementary Table 1: Comparative analysis of the most highly differentiated miRNA in tissues from MIBC patients who were responders vs non-responders to chemotherapy. Highlighted in grey are the ones that were reanalyzed by qPCR.

SystematicName	Fold change([responders] vs [Non-responders])	Regulation([Yes] vs [No])
hsa-miR-24	5.1154265	up
hsa-miR-214	4.57229	up
hsa-miR-193a-3p	4.4110894	up
hsa-let-7a	4.208447	up
hsa-miR-15a	4.091291	up
hsa-let-7g	3.8028967	up
hsa-let-7c	3.746715	up
hsa-miR-130a	3.6430833	up
hsa-let-7f	3.6333504	up
hsa-miR-106b	3.5025506	up
hsa-miR-23a	3.4677122	up
hsa-miR-15b	3.446	up
hsa-miR-210	3.3543355	up
hsa-miR-21	3.2745962	up
hsa-miR-107	3.2666154	up
hsa-miR-29b	3.260668	up
hsa-miR-199a-3p	3.2600694	up
hsa-let-7d	3.2064848	up
hsa-miR-424	3.2038114	up
hsa-miR-200a	3.1746733	up
hsa-let-7e	3.1038437	up
hsa-miR-96	3.0804327	up
hsa-miR-27a	3.0731142	up
hsa-miR-26b	3.070323	up
hsa-miR-183	3.0436797	up
hsa-miR-301a	2.9929452	up
hsa-miR-26a	2.9640954	up
hsa-miR-103	2.956494	up
hsa-miR-27b	2.8818622	up
hsa-miR-23b	2.8748837	up
hsa-miR-20b	2.866379	up
hsa-miR-93	2.8621688	up
hsa-let-7i	2.8432555	up
hsa-miR-20a	2.8358867	up
hsa-let-7b	2.8347828	up
hsa-miR-363	2.7746406	up
hsa-miR-199b-5p	2.7697704	up
hsa-miR-28-5p	2.7475243	up
hsa-miR-148a	2.7208924	up
hsa-miR-17	2.6948256	up
hsa-miR-425	2.6940055	up
hsa-miR-768-3p_v11.0	2.672554	up
hsa-miR-16	2.6195066	up
hsa-miR-199a-5p	2.5988815	up

hsa-miR-203	2.591991	up
hsa-miR-22	2.5900683	up
hsa-miR-151-5p	2.568789	up
hsa-miR-29a	2.563271	up
hsa-miR-98	2.5448046	up
hsa-miR-200c	2.514143	up
hsa-miR-19b	2.508034	up
hsa-miR-455-3p	2.494452	up
hsa-miR-101	2.4267457	up
hsa-miR-331-3p	2.4092984	up
hsa-miR-34a	2.395752	up
hsa-miR-141	2.3697953	up
hsa-miR-652	2.3314195	up
hsa-miR-429	2.2713223	up
hsa-miR-30b	2.208189	up
hsa-miR-195	2.2075374	up
hsa-miR-361-5p	2.1945052	up
hsa-miR-148b	2.1562998	up
hsa-miR-140-5p	2.1513982	up
hsa-miR-454	2.1300707	up
hsa-miR-99a	2.1079018	up
hsa-miR-361-3p	2.045605	up
hsa-miR-140-3p	2.024905	up
hsa-miR-10a	2.0189683	up
hsa-miR-31*	1.9785365	up
hsa-miR-374a	1.9682323	up
hsa-miR-126	1.9664462	up
hsa-miR-324-5p	1.9492303	up
hsa-miR-29c	1.9033198	up
hsa-miR-130b	1.8911893	up
hsa-miR-142-3p	1.889597	up
hsa-miR-128	1.8813537	up
hsa-miR-25	1.8679994	up
hsa-miR-7	1.8595637	up
hsa-miR-192	1.8438987	up
hsa-miR-451	1.8307053	up
hsa-miR-196a	1.8300179	up
hsa-miR-193b	1.8177115	up
hsa-miR-200b	1.8064072	up
hsa-miR-222	1.806194	up
hsa-miR-19a	1.8035554	up
hsa-miR-30e*	1.8034444	up
hsa-miR-551b	1.7937772	up
hsa-miR-146b-5p	1.7856083	up
hsa-miR-497	1.7750026	up
hsa-miR-30c	1.7561895	up
hsa-miR-185	1.7146778	up
hsa-miR-34b*	1.7127903	up
hsa-miR-376c	1.7064095	up
hsa-miR-375	1.6957567	up
hsa-miR-95	1.6741487	up
hsa-miR-125b	1.6590105	up

hsa-miR-662	1.6583761	up
hsa-miR-221	1.6280845	up
hsa-miR-374b	1.6140921	up
hsa-miR-200b*	1.6059076	up
hsa-miR-223	1.5826814	up
hsa-miR-196b	1.5629684	up
hsa-miR-150	1.554363	up
hsa-miR-152	1.5507895	up
hsa-miR-431	1.5371733	up
hsa-miR-143	1.5202504	up
hsa-miR-10b	1.5132589	up
hsa-miR-132	1.5120989	up
hsa-miR-801_v10.1	1.5022966	down
hsa-miR-602	1.5121675	down
ebv-miR-BART12	1.5232949	down
hsa-miR-296-3p	1.5364202	down
hsa-miR-18b	1.5446181	down
hsa-miR-191*	1.5527499	down
hsa-miR-34c-5p	1.5550215	down
kshv-miR-K12-9*	1.5652902	down
hsa-miR-500	1.5922074	down
hsa-miR-636	1.5987458	down
hsa-miR-648	1.6061159	down
hsa-miR-320a	1.6291193	down
hsa-miR-885-5p	1.6384997	down
hsa-miR-1238	1.639346	down
kshv-miR-K12-3	1.639351	down
hsa-miR-512-3p	1.6497953	down
hsa-miR-877	1.6551211	down
hsa-miR-887	1.658529	down
hsa-miR-615-3p	1.6708504	down
hsa-miR-1236	1.6722618	down
hsa-miR-940	1.6756939	down
hsa-miR-155	1.6895447	down
hsa-miR-1234	1.6999598	down
hsa-miR-181d	1.7044969	down
hsa-miR-617	1.7211885	down
hsa-miR-133a	1.7223527	down
hsa-miR-202	1.7258105	down
hsa-miR-936	1.7449207	down
hsa-miR-486-5p	1.7501721	down
hsa-miR-638	1.7539074	down
hsa-miR-769-3p	1.7564	down
hsa-miR-525-5p	1.7644101	down
hsa-miR-139-3p	1.7741019	down
hsa-miR-874	1.7757512	down
hsa-miR-1225-3p	1.8155075	down
hsa-miR-10b*	1.8201276	down
hsa-miR-596	1.8297071	down
hcmv-miR-US4	1.8307956	down
hsa-miR-760	1.8434393	down
hsa-miR-625*	1.8963245	down

hsa-miR-345	1.9095659	down
hsa-miR-572	1.9192595	down
hsa-miR-575	1.9241668	down
kshv-miR-K12-8	1.931153	down
hsa-miR-518a-5p	1.9546858	down
hsa-miR-1237	1.9633648	down
hsa-miR-328	1.9691226	down
hcmv-miR-UL70-3p	1.9938328	down
hsa-miR-18b*	2.0043454	down
hsa-miR-498	2.0043652	down
hsa-miR-371-5p	2.0184712	down
hsa-miR-125b-1*	2.0320947	down
hsa-miR-28-3p	2.045776	down
hsa-miR-630	2.0569882	down
hsa-miR-550	2.0662014	down
hsa-miR-490-5p	2.0863683	down
hsa-miR-574-5p	2.086872	down
hsa-miR-584	2.0934198	down
hsa-miR-194*	2.122592	down
hcmv-miR-UL148D	2.134274	down
hsa-miR-939	2.1549318	down
hsa-miR-632	2.15637	down
hsa-miR-206	2.1764894	down
hsa-miR-125b-2*	2.1947885	down
hsa-miR-518e*	2.2049687	down
miRNABrightCorner30	2.2131386	down
hsa-miR-610	2.213554	down
hsa-miR-1224-5p	2.2328818	down
hsa-miR-708	2.2329276	down
hsa-miR-1225-5p	2.2396448	down
hsa-miR-518b	2.2489243	down
hsa-miR-622	2.2535872	down
hsv1-miR-LAT_v10.1	2.255215	down
ebv-miR-BART13	2.2617152	down
hsa-miR-1228	2.2671669	down
hsa-miR-373*	2.270236	down
hsa-miR-1228*	2.2761433	down
hsa-miR-30b*	2.277913	down
hsa-miR-193b*	2.2829251	down
hsa-miR-302c*	2.2984126	down
hsa-miR-663	2.3019562	down
hsa-miR-639	2.306527	down
hsa-miR-296-5p	2.3128664	down
hsa-miR-150*	2.3315413	down
hsa-miR-566	2.3527558	down
ebv-miR-BART7	2.3596087	down
hsa-miR-125a-3p	2.3683126	down
hsa-miR-583	2.3865469	down
hsa-miR-338-5p	2.3949478	down
hcmv-miR-US25-1	2.431466	down
hsa-miR-483-5p	2.436037	down
hsa-let-7d*	2.4381664	down

hsa-miR-99b*	2.4805777	down
kshv-miR-K12-7	2.485184	down
kshv-miR-K12-10b	2.496572	down
ebv-miR-BHRF1-1	2.5391386	down
hsa-miR-135a*	2.5489833	down
hsa-miR-129-5p	2.5820384	down
hsa-miR-520e	2.5880218	down
hsa-miR-595	2.6206522	down
hsa-miR-557	2.6381726	down
hsa-miR-516a-5p	2.6675062	down
hsa-miR-134	2.6689427	down
hsa-miR-623	2.674544	down
hsa-miR-1226*	2.7421713	down
hsa-miR-30c-2*	2.7536569	down
hsa-miR-188-5p	2.7614481	down
hiv1-miR-H1	2.7727714	down
hsa-miR-671-5p	2.7831905	down
hsa-miR-665	2.797279	down
ebv-miR-BART17-3p	2.7979145	down
hsa-miR-526b	2.8063183	down
hsa-miR-187*	2.8906887	down
hsa-miR-520b	2.9512932	down
hsa-miR-370	2.9782224	down
hsa-miR-198	3.0114477	down
hsa-miR-424*	3.0384455	down
hsv1-miR-H1	3.0990682	down
hsa-miR-23a*	3.1172748	down
hsa-miR-765	3.2083426	down
hsa-miR-195*	3.2243376	down
hsa-miR-298	3.3316617	down
ebv-miR-BART16	3.3337893	down
hcmv-miR-US33-5p	3.4906313	down
hsa-miR-601	3.545806	down
hsa-miR-518c*	3.5828366	down
hsa-miR-149*	3.7758307	down
hsa-miR-422a	3.7929358	down
hsa-miR-508-5p	3.9209347	down
hsa-miR-659	4.073046	down
hsa-miR-30c-1*	4.319919	down
